# Calcium-sensing receptor silencing in colorectal cancer is associated with promoter hypermethylation and loss of acetylation on histone 3

**DOI:** 10.1002/ijc.28856

**Published:** 2014-04-02

**Authors:** Irfete S Fetahu, Julia Höbaus, Abhishek Aggarwal, Doris M Hummel, Samawansha Tennakoon, Ildiko Mesteri, Sabina Baumgartner-Parzer, Enikő Kállay

**Affiliations:** 1 Department of Pathophysiology and Allergy Research, Medical University of ViennaVienna, Austria; 2 Department of Pathology, Medical University of ViennaVienna, Austria; 3 Department of Internal Medicine III, Medical University of ViennaVienna, Austria

**Keywords:** calcium-sensing receptor, DNA methylation, histone acetylation, histone methylation, colorectal cancer

## Abstract

The calcium-sensing receptor (CaSR) is suggested to mediate the antiproliferative effects of calcium in colon. However, in colorectal cancer (CRC) the expression of the CaSR is silenced and the underlying mechanisms leading to its loss are poorly understood. We investigated whether loss of the CaSR expression in colorectal tumors is caused by DNA hypermethylation and imbalance of transcriptionally permissive/repressive histone alterations. We observed significantly lower *CaSR* mRNA expression (*n* = 65, *p* < 0.001) in colorectal tumors compared with the adjacent mucosa from the same patient. Immunofluorescence staining confirmed downregulation of the CaSR protein also. The *CaSR* promoter was methylated to a greater extent in tumors compared with adjacent mucosa as determined by bisulfite sequencing (*n* = 20, *p* < 0.01) and by pyrosequencing (*n* = 45, *p* < 0.001), and methylation correlated inversely with mRNA expression (*n* = 20, *ρ* = −0.310, *p* < 0.05 and *n* = 45, *ρ* = −0.588, *p* < 0.001). Treatments with 5-aza-2′-deoxycytidine (DAC), a DNA methyltransferase inhibitor and/or with two different histone deacetylase inhibitors, trichostatin A (TSA) or suberoylanilide hydroxamic acid (SAHA) restored the expression of CaSR in colon cancer cells. Restored CaSR expression in Coga1A and HT29 cells was functional. Inhibition of lysine-specific demethylase 1 (LSD1) to prevent demethylation of mono- and dimethylated H3K4, increased CaSR expression only marginally. Our data show that hypermethylation of the *CaSR* promoter and H3K9 deacetylation, but not H3K4me2 demethylation are important factors that cause silencing of the CaSR in colorectal cancer.

Epidemiological studies demonstrate an inverse correlation between calcium intake and the risk of tumor development in the human colon.^1^,^2^ Chemopreventive actions of calcium rely on its ability to bind toxic secondary bile acids and ionized fatty acids, thereby neutralizing them,^3^ and on its capacity to stimulate cell differentiation, induce apoptosis, and reduce proliferation.^4^,^5^ The molecular mechanisms of the proposed direct actions of calcium in colonocytes are not well understood. Several studies have suggested that the calcium-sensing receptor (CaSR) plays an important role in this process.^6^–^11^ The CaSR is a G protein-coupled receptor, expressed by tissues involved in the control of Ca^2+^ homeostasis, including parathyroid, kidney, small intestine, and bone. The receptor is also expressed in “non-homeostatic tissues,” including colon, nervous system, breast, and heart in which its roles are not yet fully defined.^12^–^14^

In colon, *CaSR* is a putative tumor suppressor gene mediating, at least in part, the antiproliferative and prodifferentiating actions of calcium.^14^,^15^ Antiproliferative effects of Ca^2+^ are impaired or lost in colon cancer,^16^,^17^ and this could be due to reduced CaSR expression in the tumors.^18^,^19^ A recent study on tissue-specific knockout mice showed that mice lacking *CaSR* in the intestine had hyperproliferative colonic epithelial cells.^9^ Furthermore, the colons of mice that were double null for *CaSR* and *PTH* genes developed spontaneously aberrant crypt foci (ACF), and treatment with the carcinogen azoxymethane increased further the number of ACF/cm^2^ compared with wild type control mice.^20^ Identifying the mechanisms that underlie CaSR downregulation could aid the development of better strategies for the prevention and/or treatment of colon cancer.

Gene silencing due to epigenetic modifications is frequent in cancer.^21^ In normal cells, DNA methylation affecting cytosines in CpG dinucleotides ensures proper regulation of gene expression^22^, and occurs in intergenic regions and repetitive genomic sequences to maintain a transcriptionally inactive state.^23^ In gene promoters CpGs are clustered in small DNA stretches called CpG islands (CGI), which are found in 60% of promoter regions, and are rarely methylated in normal cells.^21^,^24^ Histones are major protein components of chromatin that undergo posttranslational modifications (*e.g*., acetylation, methylation, and phosphorylation).^25^ In epigenetically silenced genes in cancer, hypermethylation of CGIs is often associated with loss of acetylation on histone 3 (H3) and histone 4 (H4), loss of methylation of lysine (K) 4 on H3 (H3K4), and gain of methylation of K9 and K27 on H3 (H3K9 and H3K27), suggesting coordinated control of DNA methylation and histone modifications.^25^ At a given promoter, the marks arising from DNA methylation and histone modifications determine whether the chromatin is in an open (active) or a closed (repressed) state.^26^

The human *CaSR* gene is located in the chromosomal region 3q13.3–21. It contains six protein-encoding exons (2–7) and two 5′-untranslated exons (1A and 1B). The expression of *CaSR* is under the control of two promoters, yielding alternative transcripts differing in their 5′-untranslated regions (UTRs), but encoding the same protein.^27^,^28^ The upstream promoter contains a TATA box and a CAAT box, and harbors very few and sporadic CpGs, whereas the downstream promoter lies in a GC rich region that could be regulated by methylation.^27^,^28^

Up-to-date there is lack of knowledge regarding the methylation status of *CaSR* promoter 2 in colorectal tumors. Therefore, in the present study, we studied the entire CGI located in the second promoter of CaSR and investigated whether DNA hypermethylation and histone modifications, particularly acetylation and methylation of lysines on H3, play a role in silencing the *CaSR* in colorectal cancer.

## Material and Methods

### Materials and reagents

DMEM, FBS, hepes, l-glutamine, penicillin and streptomycin were obtained from Life Technologies (Vienna, Austria). 5-aza-2′-deoxycytidine (DAC), Trichostatin A (TSA), suberoylanilide hydroxamic acid (SAHA), Tween-20, Triton-X, and proteinase K were purchased from Sigma Aldrich (Vienna, Austria). The bisguanidine polyamine analog that inhibits LSD1 (LSD1i) was obtained from Merck Millipore (Darmstadt, Germany). NPS-2143 was obtained from Tocris (Bristol, UK). NPS-R568 was a kind gift from Dr. Arthur Christopoulos. POWER SYBR GREEN Mastermix, PureLink Quick Gel Extraction Kit, PureLink Quick Plasmid Miniprep Kit, and Topo TA Cloning Kit were purchased from Life Technologies. EpiTect Bisulfite Kit and HotStarTaq DNA Polymerase were from Qiagen (Hilden, Germany). Total human RNA was obtained from Clontech (Saint-Germain-en-Laye, France). Polyclonal anti-H3K4me2 and anti-H3K9ac antibodies were obtained from Diagenode (Liège, Belgium). Polyclonal anti-CaSR antibody was from AnaSpec (Seraing, Belgium), monoclonal anti-CaSR and anti-IgG antibodies were purchased from Abcam (Cambridge, UK). Dylight 549 anti-IgG antibody was obtained from Vector Laboratories (Peterborough, UK), DAPI was from Roche (Vienna, Austria), goat serum was obtained from Jackson ImmunoResearch (Suffolk, UK), and Fluoromount-G was purchased from Southern Biotech (Alabama, USA).

### Patients and tumor samples

Colon tumor and adjacent mucosa samples were collected from 65 CRC patients after written informed consent at the General Hospital of Vienna and the Rudolfsstiftung Hospital (Table1). This study was approved by the local ethics committee. The tumors were examined, graded, and categorized according to the TNM system by a pathologist. The adjacent mucosa was taken from the resection margin. Based on histological examination the pathologist excluded tumor infiltration. Samples were either snap frozen in liquid N_2_ or fixed in formalin and embedded in paraffin.

**Table 1:** Clinicopathological characteristics of study patients

Age(years)	
Mean ± SD	68.7 ± 12.7
**Gender** (*n*)	
Female	33
Male	32
**Location of primary tumor** (n)	
Cecum/ascending/transverse colon	24
Descending/sigmoid colon	24
Rectum	17
**Histological grade** (n)	
I	1
II	60
III	4
**Tumor staging** (n)	
1	2
2	12
3	44
4	7
**Lymph node infiltration** (n)	
Yes	32
No	31
Unknown	2

*n*: number of patients.

### Cell lines

Coga1A, Coga13, Caco2/AQ, and HT29 were maintained in DMEM containing 10% FBS, 10 mM hepes, 2 mM l-glutamine, and 100 U ml^−1^ penicillin/streptomycin at 37°C, in a humidified atmosphere of 5% CO_2_. The adenoma cell line LT97 was cultured as described previously.^29^ Mycoplasma PCR tests were routinely performed using Mycoplasma Detection Kit from VenorGem (Minerva Biolabs, Berlin, Germany). All cell lines were tested for their authenticity at DNA Diagnostic Center (London, UK).

### In vitro treatments

After reaching 30% confluency, cells were treated with 1 µM 5-aza-2′-deoxycytidine (DAC in PBS) for 72 hr. This was followed by treatment either with 100 nM Trichostatin A (TSA in DMSO), 3 µM suberoylanilide hydroxamic acid (SAHA in DMSO), 10 µM bisguanidine polyamine analog (LSD1i in bidistilled H_2_O), or a combination of these inhibitors for 24 hr. Additionally, for growth rate studies, cells were preincubated with either 1 µM NPS-R568 (in DMSO) or 1 µM NPS-2143 (in DMSO) for 30 min, before exposure to calcium (2 mM) for 48 hr. Vehicle treated cells were used as controls.

### RNA isolation, reverse transcription, and real time qRT-PCR

RNA isolation, reverse transcription, and real time qRT-PCR analysis were performed as described previously.^30^,^31^ Data were normalized to the expression of reference genes: β-actin (for patient samples) and β-2-microglobulin (β2M, for cell lines), and set relative to the calibrator to calculate the ΔΔC_T_ value. cDNA from total human RNA (Clontech) was used as a calibrator. Primer sequences of *β-actin*, *β2M*, and *CaSR* were described previously.^31^–^33^

### Bisulfite-specific PCR and pyrosequencing

To identify the CpG island/s in the *CaSR* promoter region we used the following settings of Methyl Primer Express v1.0 Software (Applied Biosystems, Austria): minimum length 300 bp, maximum length 2,000 bp, C+Gs/total bases >50%, and CpGs observed/CpGs expected >0.6. The same software was used to design primers for bisulfite-specific PCR. Detailed description of primer sequences and PCR conditions are provided in Supporting Information Table 1.

DNA was isolated by digestion with proteinase K following phenol/chloroform extraction. DNA was bisulfite modified using EpiTect Bisulfite Kit. PCR amplification was performed with HotStarTaq DNA Polymerase. PCR products were run on a 2% low-melt agarose gel, bands were excised, and purified with PureLink Quick Gel Extraction Kit. Cloning was performed with the Topo TA Cloning Kit, electro-competent bacteria were used for subcloning following the recommended protocol. Bacterial cultures were grown in lysogeny broth media containing 50 µg ml^−1^ kanamycin. Minipreps were performed with PureLink Quick Plasmid Miniprep Kit and DNA sequencing was performed in Genetic Analyzer 3130xI (Applied Biosystems). For each sample multiple clones were sequenced. Results were analyzed using BiQ Analyzer software.^34^ Pyrosequencing of the *CaSR* promoter was carried out by EpigenDX, MA.

### Chromatin immunoprecipitation of H3K4me2 and H3K9ac

Abundance of dimethylated H3K4 and acetylated H3K9 bound to the *CaSR* promoter region was analyzed in seven regions. Chromatin immunoprecipitation (ChIP) was performed as described previously.^35^ The following antibodies were used: H3K4me2, H3K9ac, and control IgG at a final concentration of 1 µM. DNA was diluted to a final concentration of 100 ng ml^−1^ in bidistilled H_2_O. qPCR analyses were performed with POWER SYBR GREEN Mastermix and the data were normalized with respect to input according to the formula: 2^−ΔCp^x100, where Δ*C*_p_ is *C*_p_(input)-*C*_p_(immunoprecipitated DNA), and *C*_p_ is the fractional cycle number. The primer sequences are provided in Supporting Information Table 2.

### Immunofluorescence staining of colon cancer cells and tissue sections

After treatments, cells grown on sterile glass cover slips were fixed with 3.7% paraformaldehyde in PBS for 20 min, and washed extensively with PBS. Paraffin-embedded 5-µm tissue sections were incubated for 25 min at 60°C, deparaffinized, and rehydrated. After washing with PBS (pH 7.2), for antigen-retrieval sections were boiled in 0.05% citrate buffer. Samples were permeabilized with 0.2% Tween-20 (tissues) and 0.2% Triton-X (cells) in PBS for 20 min, and blocked with 5% goat serum in PBS for 30 min. Cells or tissue sections were incubated with rabbit polyclonal anti-CaSR antibody (1:100) for 1 hr at room temperature. Rabbit IgG was used as negative control. After extensive washing with PBS, samples were incubated with Dylight 549 goat-anti-rabbit-IgG (1:500). Nuclei were stained with DAPI (1:1,000) for 10 min and samples were mounted using Fluoromount-G. Protein expression data were confirmed using mouse monoclonal anti-CaSR antibody (1:200, data not shown). Whole slide images were acquired using TissueFAXS 2.04 (TissueGnostics, Austria).

### Growth rate of colon cancer cells

Colon cancer cells, Coga1A and HT29 were seeded in 96-well plates. After treatments cells were counted with T10 Automated Cell counter (BioRAD, CA).

### Statistical analysis

All statistical analyses were performed with SPSS version 18 and graphs were drawn with GraphPad Prism version 5. In case of non-normal distribution, data were log transformed to achieve normal distribution, and then subjected to paired *t*-test. For group comparisons we used one way ANOVA followed by Tukey's multiple posttest comparisons. Correlation analyses were performed using Spearman's correlation coefficient.

## Results

### CaSR expression is reduced in colorectal adenocarcinomas

All patients in our cohort were Caucasian, half of them were female (50.8%), the mean age was 68.7 years. 92.3% of the tumor samples were classified as moderately differentiated (G2). In the studied patient cohort, *CaSR* mRNA expression correlated with the anatomical location of the primary tumor (*ρ* = 0.257, *p* < 0.05). The *CaSR* expression was the lowest in the right colon (cecum/ascending/transverse) and the highest in the left colon (descending/sigmoid) and rectum. No correlation was observed with patient age, sex, stage, or lymph node infiltration (Supporting Information Table 3).

**Figure 1:**
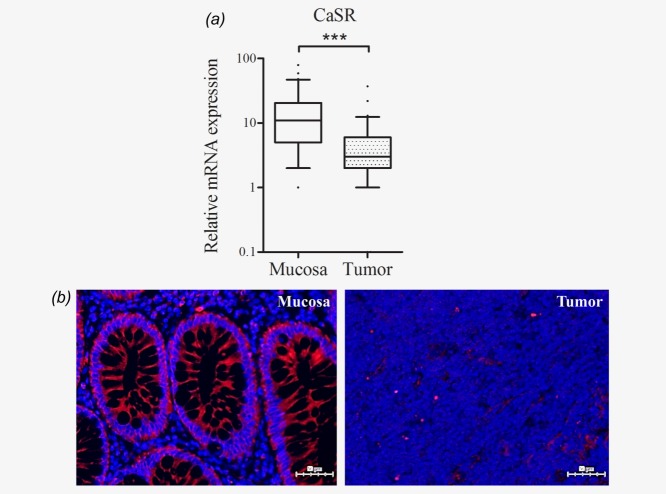
The calcium-sensing receptor is downregulated in colorectal cancer. (*a*) mRNA expression of the *CaSR* determined by real time qRT-PCR is reduced in surgical specimens of human colorectal tumors compared with their respective adjacent mucosa (*n* = 65, ***p < 0.001). (*b*) Immunofluorescence staining of paraffin embedded sections of tumors and the adjacent mucosa with rabbit polyclonal anti-CaSR antibody (red). The blue counterstain (DAPI) shows the location of nuclei. Scale bar 50 µm. [Color figure can be viewed in the online issue, which is available at wileyonlinelibrary.com.]

*CaSR* mRNA expression levels were significantly lower in tumor tissues when compared with samples of the respective adjacent mucosa (*n* = 65, *p* < 0.001; Fig. 1*a*). The CaSR protein was also barely expressed in the tumors, as confirmed by immunofluorescence staining of paraffin-embedded sections (Fig. 1*b*).

### In colorectal tumors CaSR promoter 2 is hypermethylated and methylation levels correlate inversely with mRNA expression

**Figure 2:**
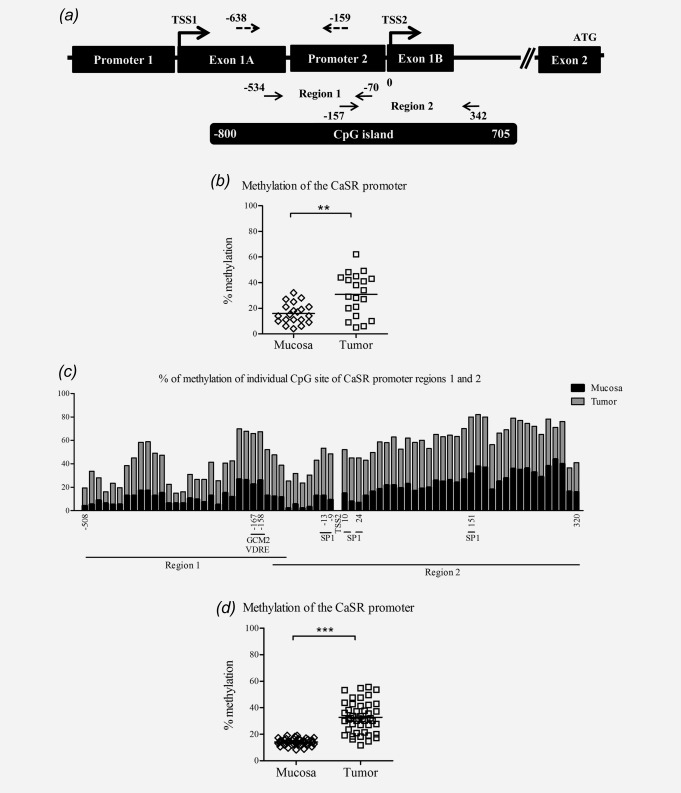
The *CaSR* promoter is hypermethylated in colorectal cancer. (*a*) Schematic illustration of the *CaSR* promoter region including the position of the CpG island, bisulfite sequencing primers (bold arrows), and region analyzed by pyrosequencing (dashed arrows). Transcription start site 2 (TSS2) according to Ref.^28^ was taken as point of reference for positioning the analyzed regions. Bisulfite sequencing (*b*) and pyrosequencing (*d*) was carried out to assess the methylation level of the *CaSR* promoter. The data are presented as the mean of methylation for each patient; the medians are indicated by the lines. The *CaSR* promoter is methylated to a greater extent in tumors compared with the adjacent mucosa (*b*: *n* = 20, **p < 0.01 and *d*: *n* = 45, ***p < 0.001). (*c*) Quantitation of results as mean % of methylated cytosines relative to the total number of CpGs in the analyzed sequences in tumors (grey bars) and adjacent mucosae (black bars). Position of the CpG sites, as well as binding sites for regulatory elements is shown [vitamin D response elements (VDRE), specificity protein 1 (SP1), and glial cells missing 2 (GCM2)].

To test the hypothesis that loss of CaSR expression is caused by DNA hypermethylation, we sequenced sodium bisulfite modified DNA from tumor and adjacent mucosa samples from colon cancer patients. We identified *in silico* a CpG island of 1505 bp (−800 to 705 relative to TSS2; TSS2 was set according to Ref.^28^), spanning a region including the end of exon 1A, the entire promoter 2, exon 1B, and 85 bp downstream exon 1B. For sequencing, we divided this large CGI into two regions: Region 1, −534 to −70 and Region 2, −157 to 342 (Fig. 2*a*).

To evaluate the level of DNA methylation of the *CaSR* promoter we used two sequencing techniques, bisulfite sequencing and pyrosequencing. Bisulfite sequencing reveals the methylation patterns of large DNA regions, whereas pyrosequencing reveals methylation levels in smaller regions, however, permitting high-throughput analysis. The *CaSR* promoter was higher methylated in samples of tumor tissue compared with the apparently normal adjacent mucosa from the same patient (*n* = 20, *p* < 0.01; Fig. 2*b*). Correlation analysis revealed a negative association between *CaSR* mRNA expression and level of DNA methylation (*ρ* = −0.310, *p* < 0.05). Methylation level in tumor and respective adjacent mucosa of individual CpG sites in regions analyzed by bisulfite sequencing is shown in Figure 2*c*. Additional 45 patients were analyzed by pyrosequencing. The analysis of 18 CpG sites (−638 to −159 bp relative to TSS2; Fig. 2*a*) in the upstream region of *CaSR* promoter 2 revealed significantly higher methylation of this region in samples of tumor tissue compared with the adjacent mucosa (*n* = 45, *p* < 0.001; Fig. 2*d*) and the inverse correlation with mRNA expression was strengthened (*ρ* = −0.588, *p* < 0.001). The methylation levels were the highest in the right colon decreasing towards rectum, and correlated inversely with the site of primary tumor and lymph node infiltration (*ρ* = −0.312 and *ρ* = −0.306, *p* < 0.05, Supporting Information Table 3). We also analyzed the methylation status of the *CaSR* promoter in tissue obtained at autopsy from a control subject with a normal colon and observed very low levels of methylation in both regions analyzed (region 1: 8% and region 2: 4%).

### *CaSR* mRNA expression and promoter 2 methylation in colon tumor cell lines

We determined mRNA expression of the *CaSR* in five colon tumor cell lines Coga1A, Coga13, Caco2/AQ, HT29, and LT97 using real time qRT-PCR. Coga1A and Coga13 are in-house cell lines and were derived from a moderately differentiated and a poorly differentiated colon tumor specimen, respectively.^36^ Caco2/AQ is a subclone of Caco-2 cells^37^ and LT97 is a cell line derived from a colorectal adenoma.^29^ We observed considerable variation in *CaSR* mRNA expression (Supporting Information Table 4), as well as *CaSR* DNA methylation levels in both regions analyzed by bisulfite sequencing. Methylation in the first region, which covers 29 CpG sites ranged from 27 to 86%, whereas in the second region, which covers 44 CpGs, methylation varied from 44 to 98% (Supporting Information Table 4).

### Impact of DNMT1, HDACs, and LSD1 inhibition on CaSR expression in colon tumor cell lines

To investigate whether inhibition of DNA methylation with the DNMT1 inhibitor DAC would increase CaSR expression, we initially treated all five colon tumor cell lines: Coga1A, Coga13, Caco2/AQ, HT29, and LT97 with 1 µM DAC for 72 hr (data not shown). DAC upregulated *CaSR* mRNA expression in some, but not all cell lines. Neither increasing DAC concentration up to 5 µM nor decreasing to 0.25 µM restored *CaSR* expression in the non-responding cell lines. In subsequent analyses, we focused on the Coga1A as representative of the responsive cells and HT29 as representative of the nonresponsive cell lines. To assess the efficiency of the treatment with DAC, we sequenced the DNA before and after the treatment. The efficiency of DAC treatment in reducing methylation was the highest in HT29 cells, in which region 1 lost 69% and region 2 lost 51% of methylation (Supporting Information Figs. 1*a* and 1*b*), although CaSR expression increased only marginally. In Coga1A cells, where DAC treatment increased CaSR expression significantly, the efficiency was much lower, with loss of methylation levels between 19 and 25% (Supporting Information Figs. 1*c* and 1*d*).

Next, we investigated the impact of DNA methylation, histone methylation, and histone acetylation on CaSR expression. Coga1A and HT29 cells were treated with the DNA methyltransferase inhibitor DAC for 72 hr, followed by 24 hr treatments with either of the histone deacetylase inhibitors TSA (100 nM) or SAHA (3 µM), and/or the LSD1 inhibitor (10 µM) to prevent demethylation of H3K4me1 and H3K4me2.

**Figure 3:**
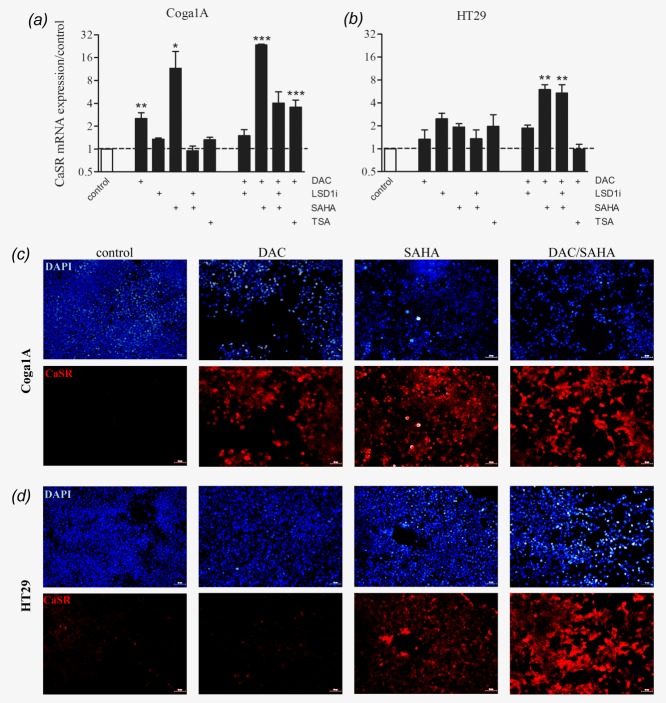
CaSR expression is restored in colon cancer cell lines upon treatments with epigenetic drugs. Coga1A and HT29 cells were treated with DAC (1 µM) for 72 hr. This was followed by treatments with TSA (100 nM) or SAHA (3 µM), and/or LSD1i (10 µM) for 24 hr. (*a*, *b*) CaSR mRNA expression and (*c*, *d*) protein expression after treatments with epigenetic drugs. Immunofluorescence staining of the CaSR protein (red), the blue counterstaining (DAPI) shows the location of nuclei. Bars represent mean ± SEM of three to five independent experiments, asterisks above bars indicate statistically significant changes compared with the vehicle control, *p < 0.05, **p < 0.01, ***p < 0.001. Scale bar in immunofluorescence images 50 µm. [Color figure can be viewed in the online issue, which is available at wileyonlinelibrary.com.]

In Coga1A cells (Fig. 3*a*), we observed a significant upregulation of *CaSR* mRNA expression in the groups that were treated with DAC and DAC/TSA compared with the vehicle control (*p* < 0.01 and *p* < 0.001). Treatment with SAHA or DAC/SAHA also resulted in a significant induction of *CaSR* mRNA expression (*p* < 0.05 and *p* < 0.001).

In HT29 cells (Fig. 3*b*), none of the drugs induced CaSR expression when applied alone. We observed an induction in *CaSR* mRNA expression only in the groups treated with the combinations DAC/SAHA (*p* < 0.01) and DAC/SAHA/LSD1 inhibitor (*p* < 0.01). Immunofluorescence staining confirmed upregulation of the CaSR protein in the cells treated with the epigenetic modifying drugs (Figs. 3*c* and 3*d*, and Supporting Information Figs. 2*a* and 2*b*).

**Figure 4:**
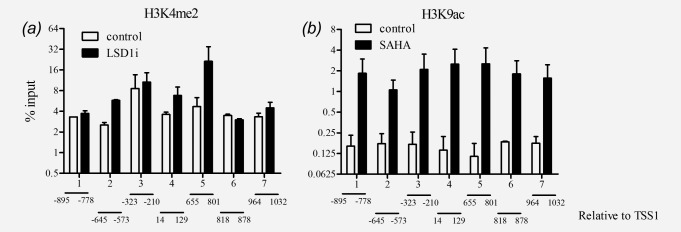
Occupancy of the *CaSR* promoter by H3K4me2 and H3K9ac. Coga1A cells were treated with 10 µM LSD1i or 3 µM SAHA for 24 hr. Presence of H3K4me2 and H3K9ac was analyzed by chromatin immunoprecipitation. After treatments with LSD1i and SAHA, dimethylation of H3K4 was partially restored (*a*), while acetylation of H3K9 was restored at all analyzed sites (*b*). Bars represent mean ± SEM of three independent experiments.

To confirm that treatment with HDAC and LSD1 inhibitors affected the histones linked to the *CaSR* promoter we performed chromatin immunoprecipitation (ChIP) using antibodies against H3K4me2 and H3K9ac. The seven regions that were analyzed span a sequence of 1927 bp (−895 to 1032 relative to TSS1; TSS1 was set according to Ref.^28^) and encompass both *CaSR* promoters. We observed enrichment in H3K4me2 only in the regions 2, 4 and 5 of *CaSR* promoter in the cells treated with the LSD1 inhibitor compared with the vehicle control (Fig. 4*a*). We next determined whether treatment with SAHA, an inhibitor of HDACs would increase H3K9ac levels in the *CaSR* promoter. In all seven regions analyzed, ChIP showed acetylation of H3K9 after SAHA treatment compared with the vehicle control, confirming that upregulation of CaSR expression was dependent on acetylation of H3K9 in the *CaSR* promoter (Fig. 4*b*).

### Restored CaSR is functional

To study whether the restored CaSR protein is functional, we performed growth rate studies. If the CaSR mediates the antiproliferative effects of calcium, increasing expression of the receptor should enhance the responsiveness to CaSR ligands. To ensure that the cellular responses observed are dependent on the CaSR we used positive and negative allosteric modulators of the receptor. Cells treated previously with 1 µM DAC for 72 hr, followed by 24 hr treatment with 3 µM SAHA were incubated for 30 min with either 1 µM NPS-R568 or 1 µM NPS-2143, and then exposed to 2 mM calcium for 48 hr.

**Figure 5:**
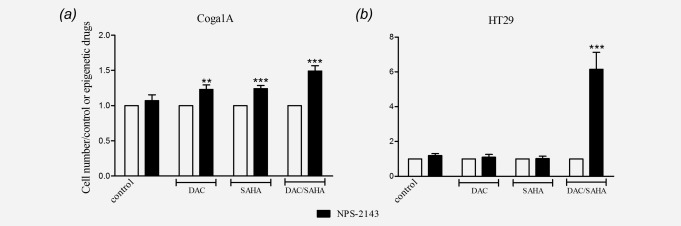
Restored CaSR is functional. Coga1A and HT29 cells were treated with 1µM DAC for 72 hr and/or 3µM SAHA for 24 hr. Cell were then pretreated for 30 min with 1 µM NPS-2143, followed by treatment with 2 mM calcium for 48 hr, after which cells were counted. Data were plotted against vehicle control or corresponding epigenetic treatment. Bars represent mean ± SEM of three independent experiments, asterisks above bars indicate statistically significant changes compared with the respective epigenetic treatment, *p < 0.05, **p < 0.01, ***p < 0.001.

In Coga1A cells, inhibition of CaSR by NPS-2143 led to increased proliferation in all the groups where treatment with the epigenetic drugs increased CaSR expression (Fig. 5*a*). This effect was more prominent in the groups treated with SAHA or DAC/SAHA that had higher induction of CaSR expression (*p* < 0.001) than in the group treated with DAC alone (*p* < 0.01). In the groups where the CaSR expression was not restored, NPS-2143 had no effect.

In HT29 cells (Fig. 5*b*) only the combined treatment with DAC/SAHA induced significantly CaSR expression, as a consequence addition of the CaSR inhibitor NPS-2143 restored proliferation only in this group (*p* < 0.001).

To further confirm the functionality of the re-expressed CaSR after the treatments with DAC/SAHA, we exposed both cell lines to the positive allosteric modulator of the CaSR NPS-R568. Treatment with NPS-R568 resulted in significantly decreased cell proliferation in Coga1A and HT29 cell lines (Supporting Information Figs. 3*a* and 3*b*).

## Discussion

In this study, we investigated which epigenetic mechanisms would control CaSR expression in colorectal cancer. In tumor samples from colon cancer patients we found higher percentages of methylated CpG sites in the *CaSR* promoter 2 when compared with samples of apparently normal adjacent mucosa. Since the methylation level correlated inversely with *CaSR* mRNA expression, we assume that one of the causes of CaSR loss in colorectal cancer is DNA hypermethylation. We found that reduced acetylation of lysine 9 on histone 3 also contributes to CaSR silencing. Methylation status of histones seems to have less impact. For instance, inhibition of the histone demethylase LSD1, which demethylates H3K4me1 and H3K4me2,^38^ could not restore CaSR expression.

The loss of CaSR expression in colorectal cancer has been reported previously,^18^,^19^ however, the causes of this downregulation are not well understood. The *CaSR* gene has a large CpG island (CGI) in promoter 2,^27^,^28^ therefore, we hypothesized that methylation of this promoter could be a key determinant of CaSR expression and its silencing in colon cancer. A previous study reported a possible role for DNA hypermethylation of exon 1A in regulation of CaSR expression.^19^ Surprisingly, the impact of DNA methylation of the second promoter, which harbors a larger number of CpGs, was not considered. A recent study in neuroblastomas, showed that DNA hypermethylation of the CGI in *CaSR* promoter 2 impairs CaSR expression in tumors with *MYCN* amplification and is predictive of poor clinical outcome.^39^ However, another study in parathyroid adenomas suggested that reduced CaSR expression is independent of exon 1A methylation, indicating that other mechanisms might be involved in loss of CaSR expression.^40^ Methylation in immediate proximity to TSSs may block initiation of transcription,^41^ therefore, in the current study we analyzed the role of methylation across the entire promoter region 2, including elements of the *CaSR* promoter that lie between exons 1A and 1B.

In samples of tumor tissue from our patient cohort, all analyzed regions of the *CaSR* promoter 2 showed increased DNA methylation when compared with the matched samples of apparently normal adjacent mucosa. We found significant inverse correlation between methylation level of *CaSR* promoter 2 in tumor tissues and anatomical location of the primary tumor. The highest methylation was observed in the right colon. This is in line with the observation that tumors originating from different colon segments have distinct DNA methylation profile, showing gradual increase in methylation from rectum towards cecum.^42^ Surprisingly, we found negative correlation between methylation of the whole CpG island and lymph node infiltration.

In the sample from a healthy colon tissue obtained at autopsy, both regions were almost unmethylated (8 and 4%, respectively). Interestingly, in the second region, especially after TSS2 we observed enhanced methylation already in the adjacent mucosa. This suggests that methylation of the *CaSR* promoter is an early phenomenon in the process leading to colon cancer and can affect the apparently normal adjacent mucosa. Although, the histological examinations attest that the adjacent mucosa is free of cancerous cells, it seems that molecular changes leading to transformation might already have occurred. Similar results were reported also in a recent study showing that 19% of the adjacent mucosa samples carried copy-number gain of the proto-oncogene *CYP24A1*, leading to overexpression of this enzyme.^30^

Besides methylating cytosines, DNMTs may coordinate other chromatin-mediated aspects of gene expression at sites of gene promoters.^23^ Thus, recruitment of DNMT1 to promoter regions has an impact on various histone modifications.^43^ Several histone modifying enzymes, such as histone deacetylase 1 and 2 (HDAC1/2),^44^–^46^ as well as the lysine-specific demethylase 1 (KDM1A/LSD1)^47^ have been reported to interact with DNMT1.^43^ In the assessed cell lines, Coga1A and HT29, treatments with the DNMT1 inhibitor DAC, and the HDAC inhibitors SAHA and TSA resulted in induction of CaSR expression in a cell line-dependent manner. Surprisingly, in HT29 cells, the strong demethylation of the *CaSR* promoter by DAC treatment did not result in activation of transcription and re-expression of the CaSR. However, DAC potentiated the effects of SAHA. It seems that in these cells at least two epigenetic modifications are needed for the re-expression of the CaSR: DNA methylation and acetylation of H3K9, or other factors could be responsible for inhibition of CaSR expression.

It has been shown previously that *de novo* DNA methylation cannot take place on nucleosomes, in which H3K4me2 or H3K4me3 occur.^48^ A recent study has demonstrated overexpression of LSD1 in colorectal tumors.^49^ Therefore, we treated our cells with an inhibitor of LSD1^50^ to preserve H3K4me2 levels, and thereby prevent DNA methylation. However, exposure of Coga1A and HT29 cells to the LSD1 inhibitor had no effect on *CaSR* mRNA expression, suggesting that partially restored dimethylation of H3K4 was not sufficient to induce CaSR expression.

We were able to show that the re-expressed CaSR is functional based on the responses observed upon treatment with its activator NPS-R568 or inhibitor NPS-2143. These findings indicate that the epigenetic drugs are able to facilitate re-expression of a functional CaSR.

Changes in epigenetic patterns are among the major mechanisms involved in tumorigenesis. Our results strongly indicate that alterations in expression of the CaSR in colorectal tumorigenesis are dependent on a broad disturbance of epigenetic mechanisms. We found that the loss of CaSR expression is caused by DNA hypermethylation and loss of H3K9 acetylation. As the CaSR is considered to be a tumor suppressor in the colon, its loss may contribute to the pathogenesis of the disease. Lesions that have lost CaSR might have increased susceptibility to proliferative stimuli. Thus, strategies aiming to upregulate CaSR expression may be of value either in prevention and/or treatment of colorectal cancer.

## Abbreviations

CaSR: calcium-sensing receptor
CGI: CpG island
ChIP: chromatin immunoprecipitation
CRC: colorectal cancer
CYP24A1: 1,25-dihydroxyvitamin D 24-hydroxylase
DAC: 5-aza-2'-deoxycytidine
DNMT1: DNA methyltransferase 1
GCM2: glial cells missing 2
H3K4me1: monomethylated lysine (K) 4 on histone (H) 3
H3K4me2: dimethylated lysine (K) 4 on histone (H) 3
H3K4me3: trimethylated lysine (K) 4 on histone (H) 3
H3K9ac: acetylated lysine (K) 9 on histone (H) 3
HDAC: histone deacetylase
LSD1: lysine-specific demethylase 1,
MYCN: v-myc avian myelocytomatosis viral oncogene neuroblastoma derived homolog
SAHA: suberoylanilide hydroxamic acid
SP1: specificity protein 1
TSA: Trichostatin A
TSS: transcription start site
UTR: untranslated region
VDRE: vitamin D response element
