# Supplementary figures and images for: Calcium-sensing receptor silencing in colorectal cancer is associated with promoter hypermethylation and loss of acetylation on histone 3
Source: Int J Cancer. 2014 Apr 2;135(9):2014–23. doi: 10.1002/ijc.28856 (PMC4282356; doi:10.1002/ijc.28856)

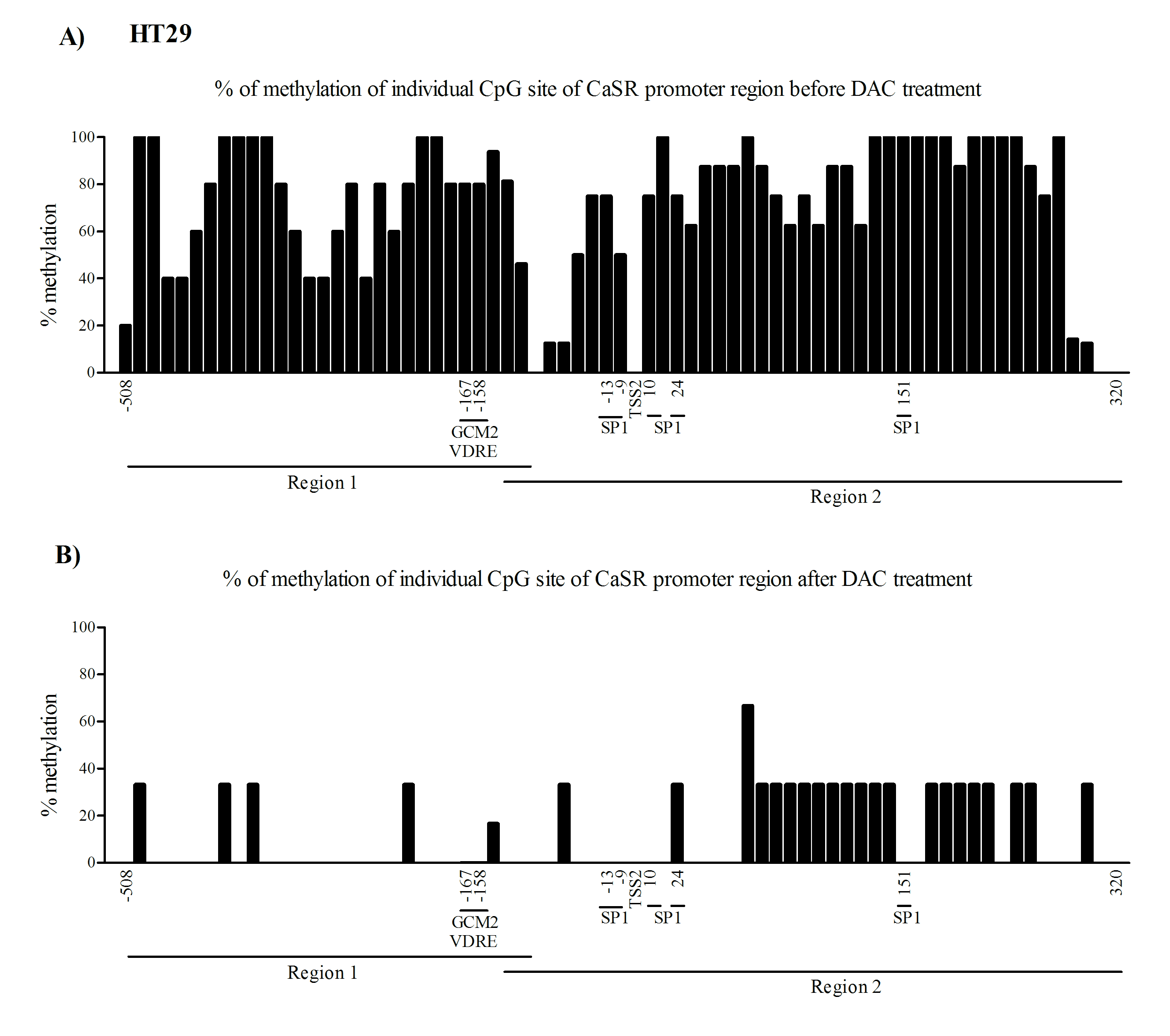

Supplement: Supplementary file 1 [file ijc0135-2014-sd1.tif]

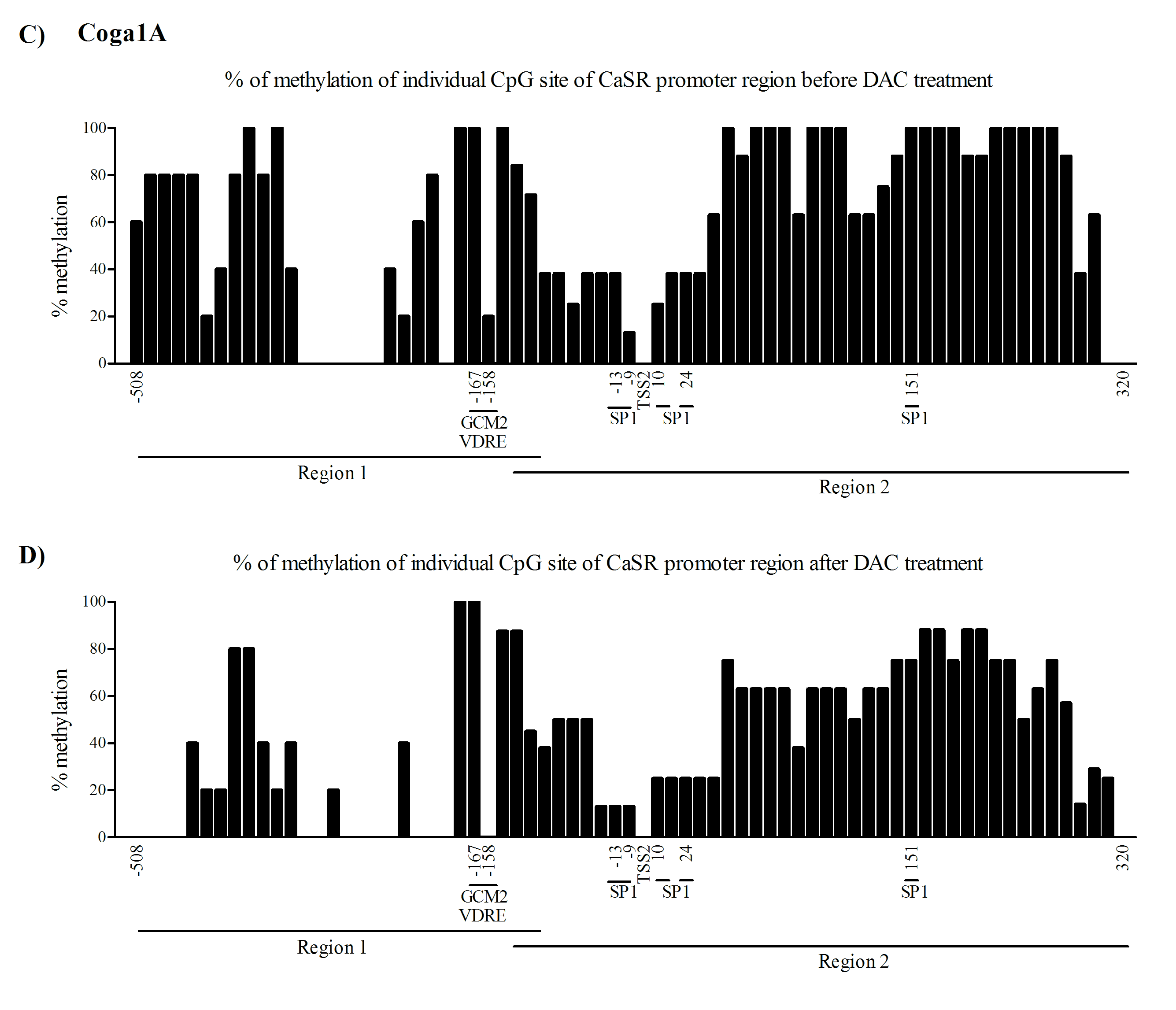

Supplement: Supplementary file 2 [file ijc0135-2014-sd2.tif]

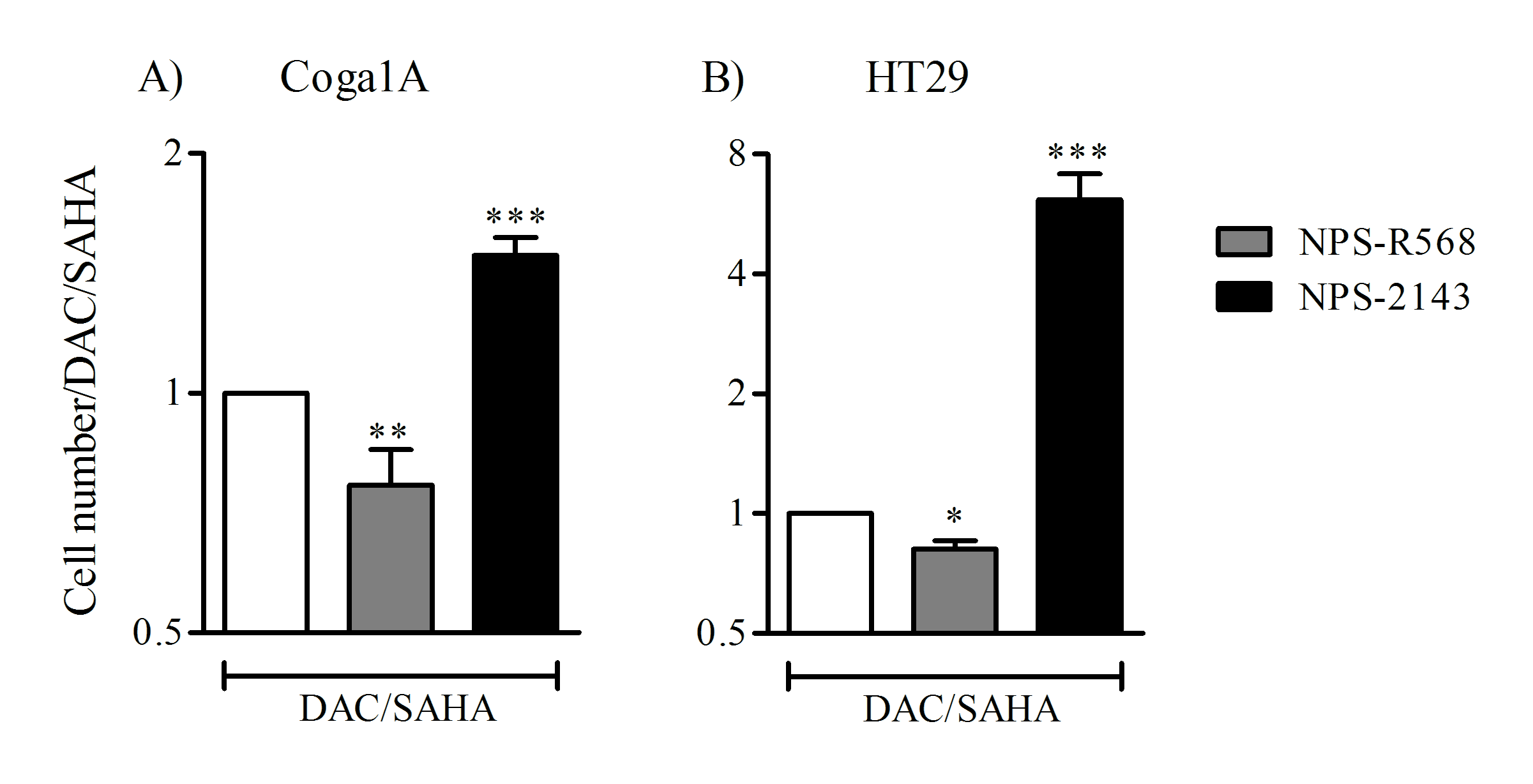

Supplement: Supplementary file 3 [file ijc0135-2014-sd3.tif]

## Slide 1
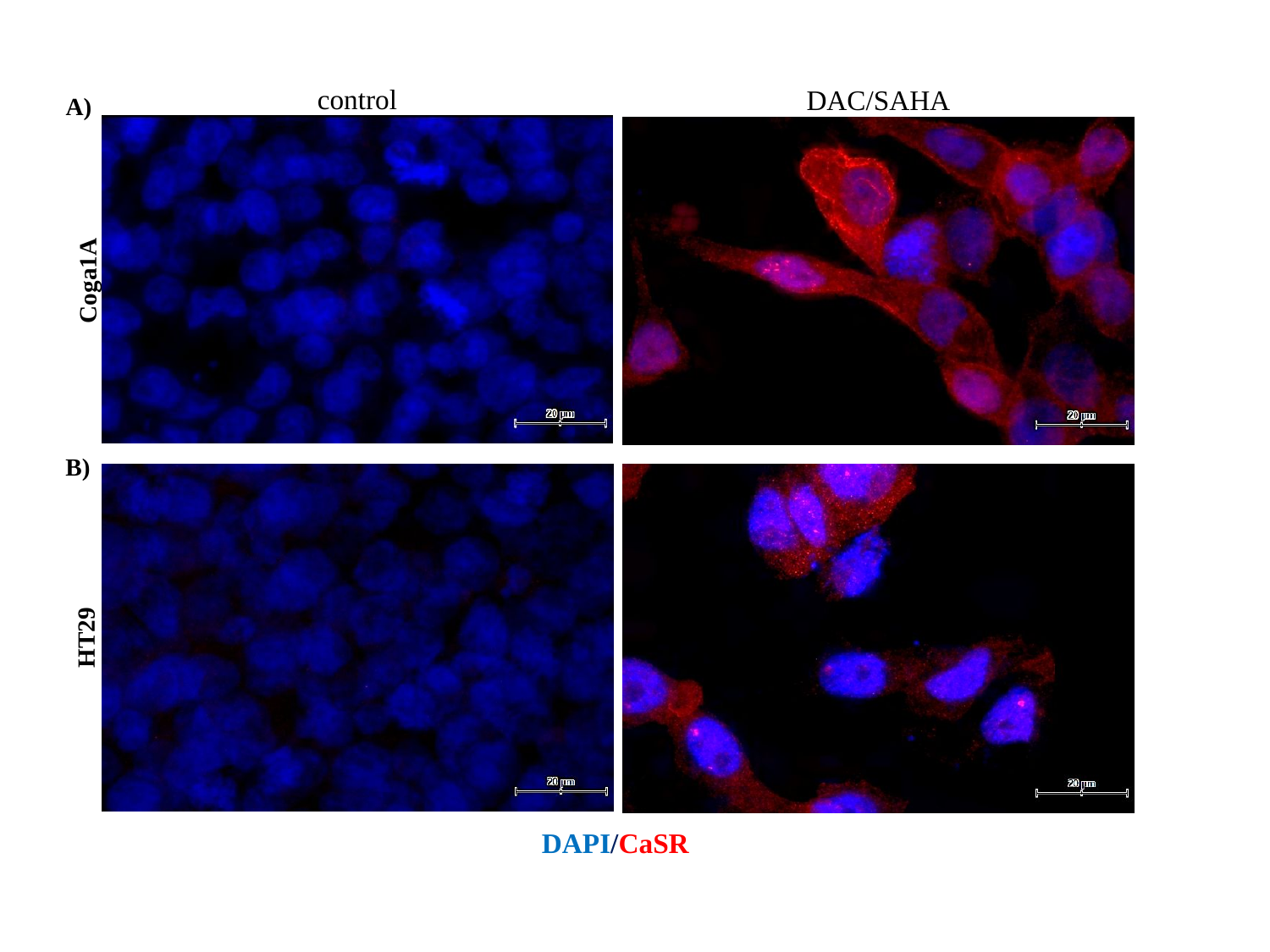

control
DAC/SAHA
Coga1A
HT29
DAPI/CaSR
A)
B)

Supplement: Supplementary file 4 [file ijc0135-2014-sd4.ppt]
