# Supplementary material for: Calcium-sensing receptor silencing in colorectal cancer is associated with promoter hypermethylation and loss of acetylation on histone 3
Source: Int J Cancer. 2014 Apr 2;135(9):2014–23. doi: 10.1002/ijc.28856 (PMC4282356; doi:10.1002/ijc.28856)
Supplement: Supplementary file 5 [file ijc0135-2014-sd5.doc]

**Supplementary Tables**

**Supplementary Table 1.** Primer sequences and conditions used for bisulfite-specific PCR for analysis of methylation in the CpG island in the *CaSR* promoter 2 region

| Region | **Forward** | **Reverse** | **Annealing T °C** |
| --- | --- | --- | --- |
| 1 | GGAAGGAGGGAGTTGTTTGTTAGTA | AACTACCTTCTAACCCCTACCCCAA | 62 |
| 2 | GATTTTGAAGAGTTAGTTAAGTTTTTTG | AAACCCTTAACCCCCAAACC | 56.4 |

**Supplementary Table 2.** qPCR primers used in chromatin immunoprecipitation (ChIP) assay

| Region | **Forward** | **Reverse** |
| --- | --- | --- |
| 1 | ACCACCCAATACCATGCTTC | AGAGCCAAGAGCTGAACCTG |
| 2 | CCAGGTGTAATCCGACCAGT | TCAGTTCAAAACCCGAATGA |
| 3 | TTGGCCATAATGAGGATGTG | AGTCAAGGAAAGTGCCCTGTA |
| 4 | TTGAAGCACTTCTGTCTTGATG | AAAGCAAACAGAGCCAAACTG |
| 5 | GGCACGCGATTTGTATTTATT | TTCCCTGACCCCTACTCCTT |
| 6 | GATTTGAGAGCCGGGAAC | GCGCTTAGGTCCTCTCCT |
| 7 | TAAGCTCTCTGCGGGCTAAG | CTGCCTTCTAACCCCTACCC |

**Supplementary Table 3.** Correlation of *CaSR* mRNA and methylation levels with clinocopathological features of study patients

|  | *CaSR* mRNA (ρ) | p value | *CaSR* methylation (ρ) | p value |
| --- | --- | --- | --- | --- |
| **Age** | 0.074 | ns | 0.047 | ns |
| **Gender** | -0.016 | ns | 0.086 | ns |
| **Location of primary tumor** | 0.257 | <0.05 | -0.312 | <0.05 |
| **Tumor staging** | -0.058 | ns | -0.154 | ns |
| **Lymph node infiltration** | -0.029 | ns | -0.306 | <0.05 |

ρ: Spearman’s correlation coefficient

**Supplementary Table 4.** *CaSR* mRNA expression and promoter methylation levels in colon tumor cell lines

| Cell lines | mRNA expression  (2^-ΔΔCT^ and range) | Methylation (%) | |
| --- | --- | --- | --- |
|  |  | Region 1 | Region 2 |
| Coga13 | 2.1 (1.9-2.4) | 77 | 98 |
| LT97 | 3.7 (3.2-4.5) | 27 | 44 |
| HT29 | 4.2 (3.6-4.9) | 75 | 68 |
| Coga1A | 4.4 (3.7-5.4) | 55 | 72 |
| Caco2/AQ | 7.6 (6.6-8.8) | 86 | 87 |
